# The Cytogenetics of the Water Buffalo: A Review

**DOI:** 10.3390/ani11113109

**Published:** 2021-10-30

**Authors:** Alessandra Iannuzzi, Pietro Parma, Leopoldo Iannuzzi

**Affiliations:** 1 National Research Council (CNR), Institute of Animal Production System in Mediterranean Environment (ISPAAM), Piazzale E. Fermi, 1, 8055 Portici, Italy; alessandra.iannuzzi@cnr.it; 2 Department of Agriculture and Environmental Sciences, University of Milan, 20133 Milan, Italy; pietro.parma@unimi.it

**Keywords:** chromosome abnormality, evolution, molecular cytogenetics, reproduction, water buffalo

## Abstract

**Simple Summary:**

Currently, there are two recognized genera of buffalo worldwide: the *Syncerus* (from the African continent), and the *Bubalus* (from the southwest Asian continent, Mediterranean area, southern America, and Australia). All species belonging to these genera have specific chromosome numbers and shapes. Because of such features, the study of chromosomes is a fascinating biological basis for differentiating the various species (and hybrids) of buffaloes and characterizing their karyotypes for evolutionary, clinical, and molecular studies. In this review, we report an update of the most important studies in which the evolutionary, clinical, and molecular cytogenetics of buffaloes were described—particularly those belonging to the river buffalo. In addition, we show new data on swamp buffalo chromosomes.

**Abstract:**

The water buffalo (*Bubalus bubalis*), also known as the Asian buffalo, is an essential domestic bovid. Indeed, although its world population (~209 million heads) is approximately one-ninth that of cattle, the management of this species involves a larger human population than that involved with raising cattle. Compared with cattle, water buffalo have been understudied for many years, but interest in this species has been increasing, especially considering that the world population of these bovids grows every year—particularly that of the river buffalo. There are two genera of buffalo worldwide: the *Syncerus* (from the African continent), and the *Bubalus* (from the southwest Asian continent, Mediterranean area, southern America, and Australia). All species belonging to these two genera have specific chromosome numbers and shapes. Because of such features, the study of chromosomes is a fascinating biological basis for differentiating various species (and hybrids) of buffaloes and characterizing their karyotypes in evolutionary, clinical, and molecular studies. In this review, we report an update on essential cytogenetic studies in which various buffalo species were described from evolutionary, clinical, and molecular perspectives—particularly considering the river buffalo *(Bubalus bubalis* 2n = 50). In addition, we show new data on swamp buffalo chromosomes.

## 1. Introduction

Chromosomes are an exciting biological material for studying the evolution of species, because most mutations accumulate at the chromosome level. At the same time, abnormal chromosome constitution is responsible for defects in both body conformation and reproduction. The water buffalo (*Bubalus bubalis*), also known as the Asian buffalo, is a crucial domestic bovid. Indeed, although its world population (~208,098,759 heads) [1] is approximately one-ninth that of cattle, the management to raise this species requires a larger human population than that involved in cattle rearing. The water buffalo is raised for both milk (mainly) and meat production, although there are differences between countries. In particular, milk can be consumed directly for nutrition (as occurs in East Asian countries) or transformed into products for commercial use [1]. The buffalo species has been understudied for many years compared with cattle, but the interest in this species has increased over the past two decades, and its population has been growing by 1.8 million heads/year for river buffalo, while the swamp buffalo population has decreased in the past decade, by an average of 180,000 heads/year [2]. 

**Figure 1:**
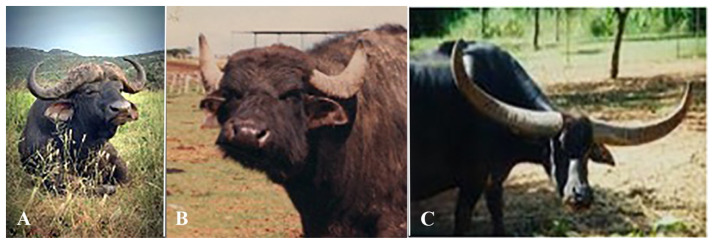
African (**A**) and Asian (**B**,**C**) buffaloes, the latter of river (**B**) and swamp (**C**) types.

There are two genera of buffalo worldwide: the African *Syncerus* (Figure 1A), and the *Bubalus*, also known as the water buffalo (Figure 1B,C). The latter is mainly present in East Asian countries, the Mediterranean area, southern America, Australia, and has several breeds—especially in India, Pakistan, and China [1]. All species belonging to these two genera have specific chromosome numbers and shapes, making the chromosomes useful for differentiating the two genera and various buffalo species (and hybrids). These chromosome changes have been the result of karyotype evolution, determining several species. Furthermore, the study of chromosomes has been mainly used in animal cytogenetics to (1) verify the relationship between chromosome abnormalities and fertility [3,4,5,6,7]; (2) physically map both type I (expressed sequences) and type II (SSRs, microsatellite marker, STSs) loci, especially using fluorescence in situ hybridization (FISH) techniques [8,9,10,11,12]; (3) correctly identify the chromosomes involved in chromosomal abnormalities via chromosome banding techniques [13,14]; (4) reveal chromosome rearrangements occurring in some chromosomal abnormalities, especially using both FISH mapping [15,16,17] and comparative genome hybridization array (aCGH) techniques [18,19]; (5) compare related and unrelated genomes by using the Zoo-FISH technique [20,21,22,23,24,25,26,27,28], centromeric SAT sequences by FISH mapping [29], or detailed FISH mapping along chromosomes [30,31,32,33,34,35,36]; and (6) test the genome stability of several bovids, including the river buffalo, with both in vitro and in vivo (natural) exposure to potential mutagens [37,38,39,40,41,42,43,44,45], or affected by limb malformations [46].

Standard chromosomal nomenclatures have been proposed for domestic bovids, including the river buffalo, where a standard karyotype was published using six different banding techniques [47]. This is the only example of a standard karyotype in any mammalian species, including humans, using six different banding techniques. However, discrepancies in the standard chromosomal nomenclatures of domestic bovids were observed when comparing the G- and R-banding patterns of some chromosomes. The differences in these standard nomenclatures were observed using both chromosome banding and FISH mapping techniques, mainly using marker chromosomes (biarmed pairs) [48,49,50]. A final agreement for a standard chromosomal nomenclature of all of the most critical domestic bovids (including the river buffalo) was found only when the G-Q and R-banding techniques were performed on cattle chromosomes by two different cytogenetic labs also using chromosome-specific markers via FISH mapping [51,52]. The same markers used in cattle chromosomes were also assigned to river buffalo, sheep, and goat chromosomes via FISH mapping [53,54], further supporting the agreement of the results obtained at the ISCNDB2000 [52].

In this review, we report the most critical data on the studies that characterized the evolutionary, clinical, and molecular cytogenetics of buffaloes—particularly those involving the water buffalo. In addition, we also show and discuss new data on swamp buffalo chromosomes.

## 2. Evolutionary Cytogenetics

The *Bovidae* are a family belonging to the *Cetartiodactyla* order, suborder *Ruminantia*. They comprise 149 different species grouped into 7 subfamilies [55]. The Bovinae comprise several species, including those in the genera *Bos*, *Bubalus*, and *Syncerus*, the latter belonging to the tribe *Bovini*, in which domesticated and undomesticated species are included [56]. The Robertsonian translocation (rob) has been the most common chromosomal mechanism characterizing the evolutionary history of many autosome chromosomes from bovid species. Still, complex chromosome rearrangements are also followed by sex chromosomes [36,57].

### 2.1. Autsosomes

**Figure 2:**
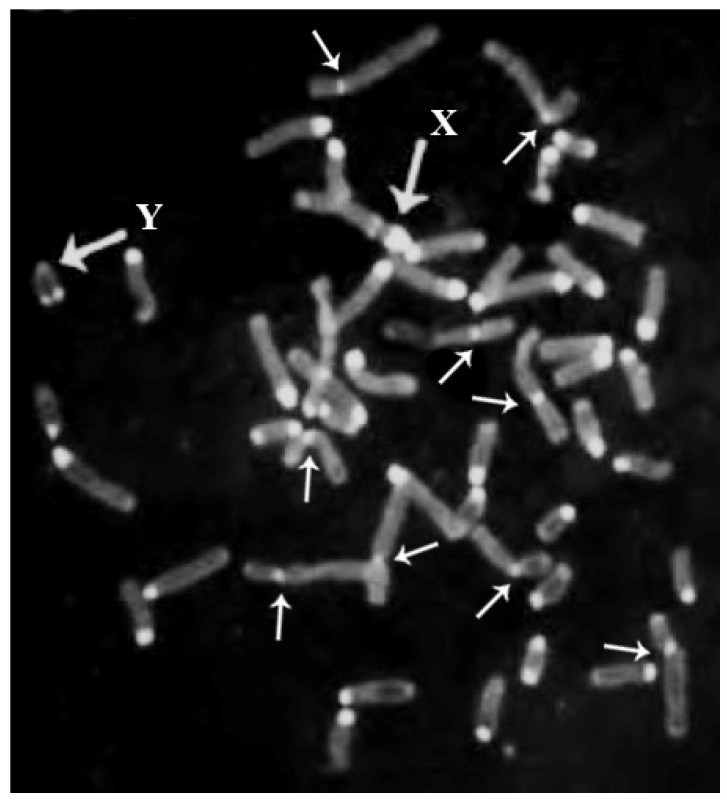
CBA banding in a river buffalo male metaphase. Note the larger HC block (C-bands) in the X chromosome also showing a proximal positive C-band. The Y chromosome has a telomeric positive C-band, making it easily distinguishable from other autosomes showing all of them to be centromeric C-band positive. Note the small amounts of HC (C-bands) in the biarmed pairs (small arrows).

Rob occurs when two non-homologous acrocentric chromosomes are joined along the centromeres. This fusion is generally accompanied by loss of constitutive heterochromatin (HC) (C-bands) localized at the centromeric regions, as shown in river buffalo [58] (Figure 2). This occurred frequently during the karyotype evolution of bovid autosomes, including the buffalo species. The number of chromosomes (2n) decreases during this process, but the number of chromosome arms (fundamental number; FN) is conserved. This simple discovery by Wurster and Benirschke [59] was later confirmed by comparative chromosome banding [60,61,62,63,64] and comparative FISH mapping analyses among bovid species [24,30,31,65,66,67,68].

With analyses of both chromosome banding comparisons and FISH mapping data, researchers have concluded that the *Bovinae* subfamily might be ancestral to the remaining subfamily (for details, see [57]). Rob occurred during the evolution of buffaloes, with their autosomal karyotypes resulting in various species having different diploid numbers, chromosome shapes, and chromosome linkage associations. All known species, subspecies, and hybrids evolved in this way, except for the buffalo *Anoa depressicornis*, which has the same 2n and FN in the two known species (Table 1).

As shown in Table 1, two genera of buffaloes exist worldwide: *Syncerus* (African buffalo), and *Bubalus* (Asian buffalo or water buffalo). The African buffalo has two subspecies: the *Syncerus caffer caffer* (2n = 52; FN = 60), and the *Syncerus caffer nanus* (2n = 54; FN = 60), with the presence of four and three biarmed pairs, respectively. By considering the biarmed pairs through the use of chromosome banding comparison with cattle chromosomes (ancestral bovid karyotype) [62] and the standard chromosomal nomenclatures [47,52], the four and three biarmed pairs of *S. c. caffer* and *S. c. nanus*, respectively, were established to be homologous to cattle chromosomes 1;13, 2;3, 5;20, and 11;29; and 1;13, 2;3, and 5;20, respectively (Table 1). For this reason, crossbreeds between the two species are possible, although the hybrids (2n = 53) could have reproductive problems because of the trivalent configurations during meiosis, and subsequent unbalanced gametes and embryos.

The Asian buffalo (BBU) includes two species: *B. bubalis*, and *B. mindorensis*. Both species are probably derived from *Bubalus arnee*, the ancestral water buffalo, which is currently considered an endangered species [1,2]. *B. bubalis* includes two subspecies: the river type (*B. b. bubalis*, 2n = 50, FN = 60), and the swamp type (*B. b. carabanensis*; 2n = 48, FN = 58) [2,69]—or simply *B. bubalis* (the river type) and *B. kerabau* (the swamp type), as recently reported [70]. Moreover, in the Asian buffalo, the diploid number varies between the two subspecies but, differently from the African buffalo—where the two subspecies differentiate from one additional centric fusion—the Asian buffaloes (*B. bubalis*) show the same number of biarmed pairs, but a different diploid number (Table 1). This phenomenon was explained by high-resolution chromosome banding comparisons between the river and swamp buffaloes [61]. Indeed, it was possible to demonstrate that river (2n = 50) and swamp (2n = 48) buffaloes are differentiated by a tandem fusion translocation that occurs between the telomeres of river buffalo chromosome 4p (BBU4p) and the centromere of river buffalo chromosome 9 (BBU9). The largest swamp buffalo chromosome 1 originated during this process [61]. This fusion was later confirmed in another study [71].

**Figure 3:**
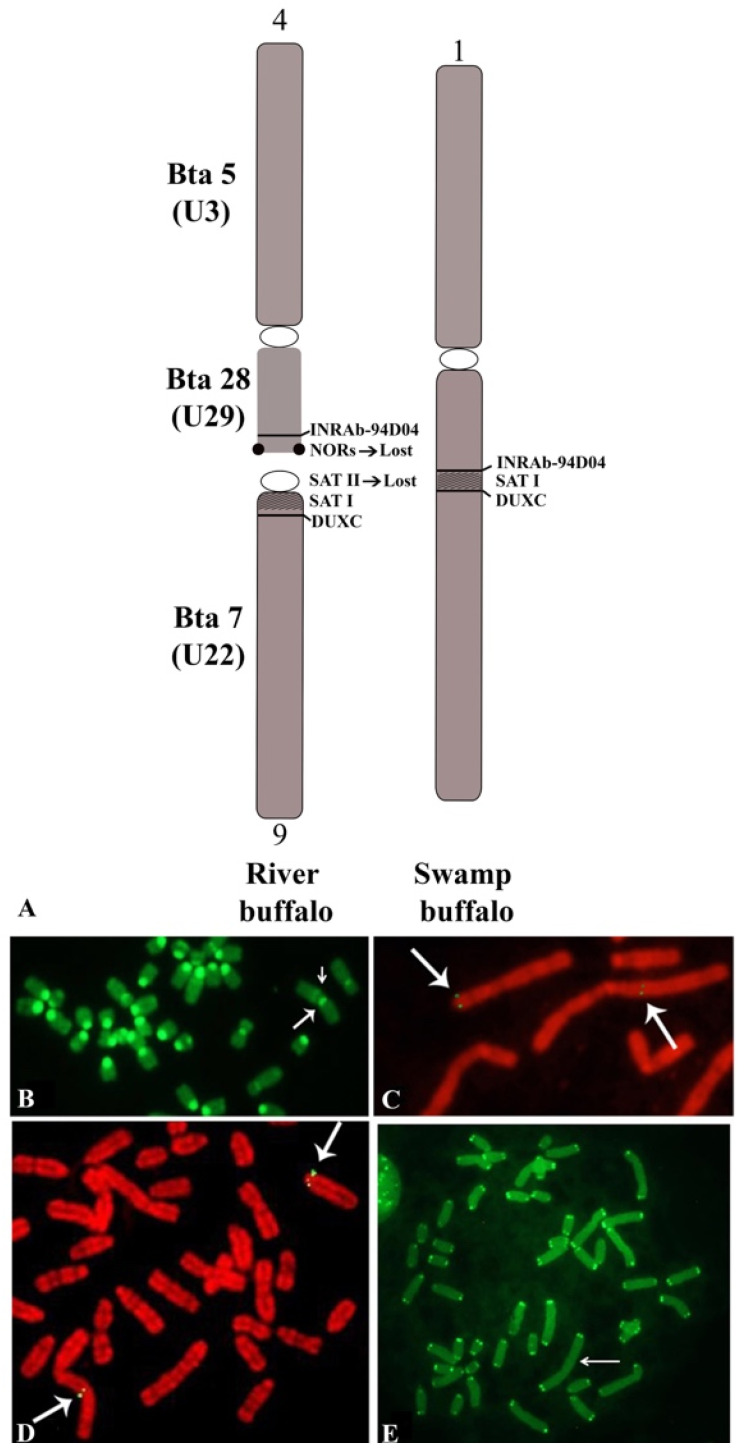
(**A**) Schematic representation of tandem fusion translocation between river buffalo chromosomes 4p (telomere) and 9 (centromere) in the origin of swamp buffalo chromosome 1. Both NORs available in BBU4p telomeres and SAT-II sequences available at the centromere of BBU9 were lost during the tandem fusion. Only the SAT-I sequences were partially conserved during the fusion. Moreover, homologous cattle chromosomes and relative syntenic groups are reported on the left of BBU4 and BBU9. (**B**) Details of CBA banding in a male metaphase plate of a hybrid river swamp buffalo. Note a large positive C-band at the centromere of swamp buffalo chromosome 1 (large arrow) and a pale and proximal positive C-band (small arrow), which are probably part of the SAT-I sequences of BBU9 conserved during the fusion. (**C**) Details of a metaphase plate of hybrid river–swamp buffaloes showing the result of the FISH mapping, with the marker INRAb_94D present both at the telomeres of BBU4 and the fusion point of swamp buffalo chromosome 1 (arrows). (**D**) Details of a metaphase plate of hybrid river–swamp buffalo showing the result of the FISH mapping, with the marker DUXC present at both the pericentromeric region of BBU9 and the fusion point of swamp buffalo chromosome 1 (arrow). (**E**) River–swamp buffalo hybrid metaphase showing the result of FISH mapping with a PNA telomeric probe. Clear hybridization signals were observed only at the telomeres of all chromosomes, and absent at the fusion point in swamp buffalo chromosome 1 (arrow).

Considering the biarmed pairs between the two subspecies, we can see that all five biarmed pairs of the river buffalo are also present in swamp buffalo. The BBU4 (biarmed) chromosome was fused with the BBU9 (acrocentric) chromosome, resulting in a large swamp buffalo chromosome 1 (Table 1). During this tandem fusion translocation, the nucleolus organizer chromosomes (NORs) available at the telomeres of river buffalo chromosome 4p, along with some centromeric sequences of river buffalo chromosome 9, were lost (Figure 3A) [61,72,73]. The visualization of the centromeric positive C-band in the large swamp buffalo chromosome 1, along with a proximal and pale positive C-band, is allowed by the CBA banding technique (Figure 3B). The proximal and pale positive band observed in swamp buffalo chromosome 1 is likely to be part of the centromere of BBU9 (SAT-I sequences). To ensure that no other chromosome regions were lost during this fusion, we used two probes mapping distally to BBU4p (homologous to BTA28) (INRA_94D04, present in the ARS UCD1.1 genome at 47 Mb of BTA28) and very proximally (pericentromeric) to BBU9 (homologous to BTA7) (DUXC, present in the ARS UCD1.1 genome at 82 Kb of BTA7) [74]. Clear hybridization signals were observed at the distal BBU4p and pericentromeric region of BBU9, as well as the corresponding regions (fusion point) of swamp buffalo chromosome 1 (Figure 3A,C,D). This result is a confirmation that only the NORs present at BBU4p telomeres and some centromeric regions of BBU9 were lost during the tandem fusion. Furthermore, it was shown by a PNA telomeric probe that there were clear signals only at the telomeres of swamp buffalo chromosome 1, but they were absent at the fusion point (Figure 3E). This fusion could have been facilitated by the presence of a positive C-band distally located—almost telomeric—at BBU4p, which is often heteromorphic within the chromosome pair, as previously reported [58].

**Figure 4:**
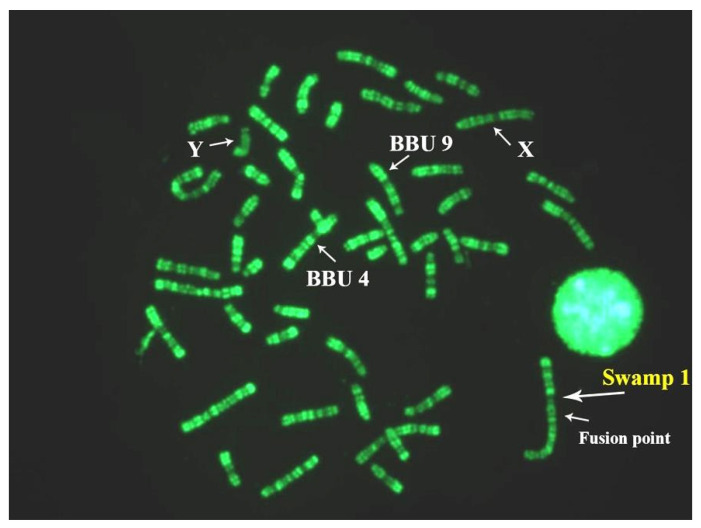
RBA-banding in a metaphase plate of a hybrid river–swamp buffalo (2n = 49). BBU4, BBU9, swamp chromosome 1, X, and Y chromosomes are indicated (arrows).

The two subspecies can interbreed, and their hybrids (2n = 49; Figure 4) are fertile. The hypothesis that the swamp buffalo was derived from the river type is supported by data, as suggested by mitochondrial DNA studies in water buffaloes [75]. However, closer genetic affinities between wild and swamp buffaloes than between wild and river buffaloes [76] have been revealed in studies on wild buffalo (*B. arnee*). Nevertheless, more information about the real natural karyotype evolution of these species can only be provided by cytogenetic studies of wild buffalo (*B. arnee*).

Crosses between river and swamp buffaloes are often undertaken to increase milk production in the swamp buffalo population, especially using Indian river buffalo breeds such as Murrah and Jafarabadi. However, the existence of riverine river–swamp hybrids with 49 chromosomes among the Indian Chilika buffalo population has been recorded for the first time. This feature was also confirmed by mitochondrial haplotype sharing between Chilika and Indian/Chinese swamp buffalo populations in a network analysis of swamp buffalo hybrids [77]. Indeed, milk production was noticeably increased by the cross between the two subspecies, although the results obtained using different breeds of both swamp and river buffalo were different (from 20% more to triple milk production just in F1 hybrids) [78,79].

Still, two issues remain crucial considering this matter: (1) the greater increase in milk production obtained from the hybrids must be sustained with adequate food nutrition, and this is often difficult considering that swamp buffaloes are raised in tiny farms with few animals (one or two heads on average) and few available food resources; (2) the hybrids have 49 chromosomes, and for this reason, reproductive problems can arise due to the presence of the trivalent configuration during meiosis, with possible subsequent anomalous meiotic segregations. In this process, abnormal gametes and embryos are produced which, consequently, can die in early embryonic life, as found in cattle carrying robs (1;29) [80,81]. Indeed, a higher frequency of abnormal pairings in the hybrids (both F1 and F2 backcrosses) at meiosis was revealed by synaptonemal complex (SC) analyses of river and swamp buffaloes and their hybrids [82].

Studies on gametes (semen in particular) and embryos using specific chromosome molecular markers and the FISH technique, as performed in a river buffalo bull carrying a chromosome abnormality via sperm FISH [83], could give more precise indications of the crosses between the two water buffalo subspecies.

**Figure 5:**
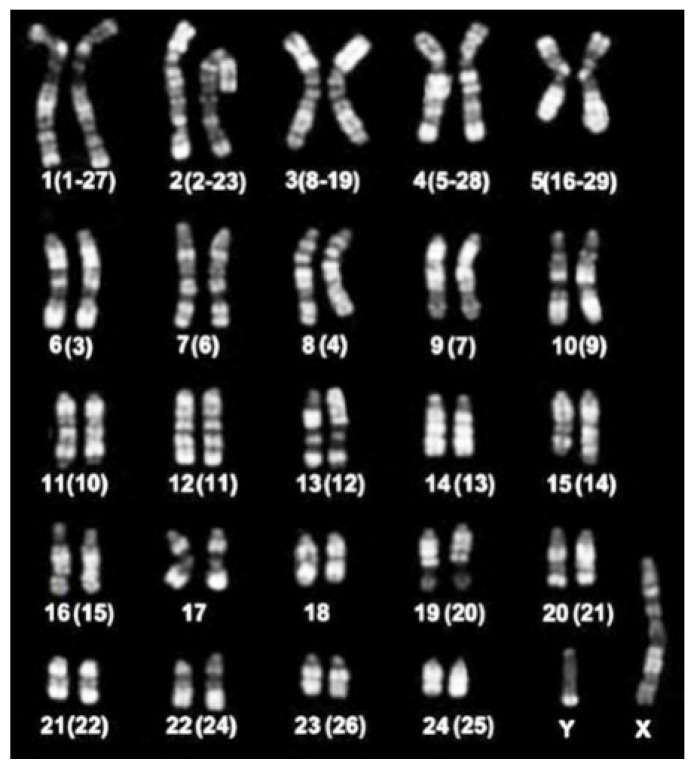
RBA-banded river buffalo karyotype is arranged according to the standard river buffalo karyotype [47]. The numbers between parentheses are referred to as the cattle homologous chromosomes, according to ISCNDB2000, 2001.

The river buffalo karyotype (2n = 50; Figure 5) has five biarmed pairs (submetacentric chromosomes). The remaining chromosomes are acrocentric, including both the X, being the largest acrocentric chromosome, and Y, the latter being one of the smallest acrocentric chromosomes. The five biarmed pairs originated via five centric fusion translocations involving the following 10 cattle (ancestral bovid) homologous chromosomes, according to both the standard river buffalo karyotype [47] and ISCNDB2000 [52]: BBU1 (1;27), BBU2 (2;23), BBU3 (8;19), BBU4 (5;28), BBU5 (16;29) (Table 1) (Figure 4). HC (C-band) losses occur along the centromeres involved in these fusions [58] (Figure 1).

In *B. mindorensis* (tamaraw) (2n = 46, FN = 56), six biarmed chromosomes resulted from centric fusions, five of which were identical to those of swamp buffalo, with one additional rob (4;14) [72] (Table 1). Therefore, *B. mindorensis* was probably derived from swamp buffalo.

In the *Bubalus* genus, we also have the anoa buffalo, with two subspecies: the *B. depressicornis depressicornis* (lowland anoa) and *B. depressicornis quarlesi* (mountain anoa), both with 2n = 48 and FN = 60 [65]. These species are the smallest buffaloes in the world. The karyotypes of these species are analogous to that of *B. bubalis*, with six biarmed chromosome pairs. Among those, four are identical to those present in *Bubalus*, the river type (BBU1, BBU2, BBU3, and BBU4), while the other two biarmed pairs of anoa are different because they involve cattle homologous chromosomes 11;20 and 17;15 [65] (Table 1). However, strong evidence is provided by phylogenetic and molecular dating analyses that the lowland anoa, river buffalo, and swamp buffalo are three distinct taxa that speciated rapidly during the Pleistocene epoch [70].

Other chromosome regions that play an essential role in the karyotype evolution of bovids are NORs. These are specific chromosome regions in which genes codifying for ribosomal RNA are intensely present. They are visualized using Ag-NOR staining [11] or FISH mapping using specific ribosomal probes [73]. The former allows visualization of only the active NORs (NORs that have organized at least one nucleolus), while the latter includes all NORs. NORs in bovids are generally available at the telomeres of autosomes—regions usually uninvolved in chromosome rearrangement (in contrast to centromeric regions). Additionally, all bovid chromosome arms were conserved. Therefore, we expected to find NORs at identical homologous chromosomes in various bovid species, but this was not the case. Indeed, NORs differ in number and chromosome position between species, especially when considering different genera [57,65]. In Table 2, the data on the NORs of the buffalo species and cattle (for comparison) are summarized. As shown, the number of NORs differs between river (with six nucleolus organizer (NO) chromosomes) and swamp (with five NO chromosomes) buffaloes. In the latter, the NORs present at the telomeres of BBU4p were lost during tandem fusion translocation originating from swamp buffalo chromosome 1, as reported above [61]. However, the remaining five NORs were conserved in the same NO chromosomes of the two subspecies. When compared with cattle, we observed that only two NORs were conserved in homologous chromosomes between cattle and water buffalo (3 and 19 of cattle corresponding to the buffalo chromosomes 6 and 3p, respectively) (Table 2). In *S. c. caffer*, only four NO chromosomes (2p, 22, 25, and 28) were found in the homologous chromosomes of the water buffalo [65], as shown in Table 2.

All species belonging to the subfamily *Bovinae* (including the *B. bubalis*) conserved the same autosomes 9 and 14, whereas in the other bovid species of the remaining subfamilies, these two chromosomes evolved via a simple translocation event from the proximal BTA9 region to the proximal BTA14 region [57,84]. In addition, some micro-rearrangements occurring among domestic bovids (including the river buffalo) have been found using a combination of bioinformatics techniques and the physical mapping of DNA markers using the FISH technique [85,86].

**Table 2:** Number of nucleolus organizer regions (NORs) and nucleolus organizer (NO) chromosomes in cattle and buffaloes. Homologous cattle NO chromosomes in buffaloes are reported between parentheses according to [52,65,87].

Species	NORs (Number of Chromosome Pairs)	NO-Chromosomes (Cattle Homologous Chromosomes Are Reported between Parentheses)
*Bos taurus* (cattle)	5	2, 3, 4, 11, 19
*Bubalus b.* (river)	6	3p (19), 4p (28), 6(3), 21(22), 23(26), 24(25)
*Bubalus b*. (swamp)	5	3p (19), 6(3), 20(22), 22(26), 24(25)
*Syncerus caffer caffer*	4	2p (3), 22(22), 25(25), 28(28)

### 2.2. Sex Chromosomes

Unlike the autosomes, the sex chromosomes of bovids evolved via complex chromosomal rearrangements, especially when comparing the *Bovinae* subfamily with the remaining members of the *Bovidae* family. Indeed, the X and Y chromosomes of domestic bovids differ in shape, size, and gene order [36,57]. In particular, the X chromosome in *Bovinae*, including the water buffalo, differs from the X chromosome in *Caprinae* by at least four chromosomal transpositions—including the centromere, with an inversion [35,57,88]. The water buffalo X chromosome is the largest acrocentric chromosome of the buffalo karyotype, and it is larger than cattle X (submetacentric) due to the presence of a large block of centromeric HC (C-band), with an additional positive C-band proximally located, whereas the cattle X chromosome is C-band negative [57]. In previous studies, it was hypothesized that the X chromosomes of cattle and river buffalo differ by pericentric inversion [62,89].

After high-resolution chromosome banding and detailed FISH mapping using several markers along the X chromosomes of both species, the gene order in the X chromosomes was the same. Still, the X chromosomes were different due to centromere repositioning (or transposition), with HC loss from river buffalo to cattle X chromosomes [35,57,88]. This finding is more likely when considering that the buffalo X chromosome is similar to the ancestral bovid karyotype [57].

Moreover, the Y chromosomes of cattle and buffaloes differ in size (more prominent in buffaloes) and shape (small submetacentric in cattle and small acrocentric in buffalo). These features occur because of a pericentric inversion differentiating the two chromosomes, with loss of HC from the buffalo to the cattle Y chromosome, as demonstrated by FISH mapping with some molecular markers [33,57]. This chromosome is generally heterochromatic (C-band positive) or has a telomeric positive C-band (Figure 2), depending on the degree of chromosomal denaturation in the C-banding technique [57]. Furthermore, the difference in size between the Y chromosomes of cattle and buffalo is only due to HC being more significant in the buffalo Y chromosome.

## 3. Clinical Cytogenetics

Clinical cytogenetics is a crucial research field of animal cytogenetics. In this research field, the study of the relationships between chromosomal abnormalities and their effects on fertility and body conformation is attempted. Since the discovery of rob (1;29) in a Swedish red cattle breed [90] and the demonstration of its deleterious effects on fertility [80,81], the clinical cytogenetics applied to domestic animals have spread throughout the world to study many domestic species, including the water buffalo. However, the water buffalo (essentially, the river buffalo) has been studied by only a few cytogenetic laboratories.

Numerical autosomal abnormalities have rarely been found in domestic animals, because the animals have abnormal body conformation. For this reason, these abnormalities are eliminated directly by the breeders. Conversely, numerical sex chromosome abnormalities are more tolerated by the species, because one of their X chromosomes is genetically inactivated [91], although some genes seem to escape gene inactivation [92]. However, numerical sex chromosome abnormalities are often correlated with sterility or low fertility, especially in females [3,4,5,6,7]. Balanced chromosomal abnormalities are generally correlated with reduced fertility due to unbalanced gametes originating during meiosis to form trivalent (centric fusions) or quadrivalent (reciprocal translocations) configurations. Subsequently, unbalanced gametes and embryos generally die in early embryonic life [3,4,5,6,7].

In Table 3, the most critical chromosomal abnormalities found so far in river buffalo and their effects on fertility are summarized. As shown in Table 3, only three autosomal chromosome abnormalities have been found in river buffaloes [93,94,95]: The first case was found in a cow with reduced fertility [93]. The second was found in a famous bull known as Magnifico [94]. A complex chromosomal rearrangement originated in both chromosomal abnormalities: fission of river buffalo chromosome 1 (BBU1) and subsequent centric fusion between chromosome BBU1p and BBU23 in the cow [93], and with BBU18 in the bull [94]. The cow had reduced fertility, producing only two lactations and calves in five years of reproductive life. One male calf had a normal karyotype, and the female carried the same translocation. After that, both females were eliminated from the reproduction farm.

The bull was famous for its high genetic value, and the use of its semen in artificial inbreeding (AI) resulted in its numerous progeny. When its chromosomal abnormality was also found in several progeny animals [94], the Italian National Breeders of Buffalo Species Association (ANASB) stakeholders decided to remove this bull from reproduction. However, in the analysis of the total and motile sperm fractions of this bull via sperm FISH, translocation was suggested to have minimal effects on the aneuploidy of its gametes. Therefore, there were also minimal effects on the reproductive abilities of the bulls [83]. These studies are demonstrations that it is crucial to obtain the banded karyotype of all bulls or males addressed to reproduction before their use as reproducers, especially in AI. The third case was reported in a Murrah buffalo bull, which had mosaicism (2n = 50, XY/2n = 50, XY, 3q-), with a partial deletion of the BBUq arm in some cells [95]. Still, the most common chromosomal abnormalities found in river buffalo involve sex chromosomes, in which mosaicism XX/XY (freemartin) constitutes the majority of cases (Table 3).

In detail, four cases of X-chromosome trisomy were found in both Murrah (two cases) and Italian Mediterranean (two cases) breeds (Table 3). In three cases, females were sterile because of severe damage to internal sex adducts [96,97,98]. In one case, the female had reduced fertility because it had only two lactations in 10 years [99] (Table 3).

**Figure 6:**
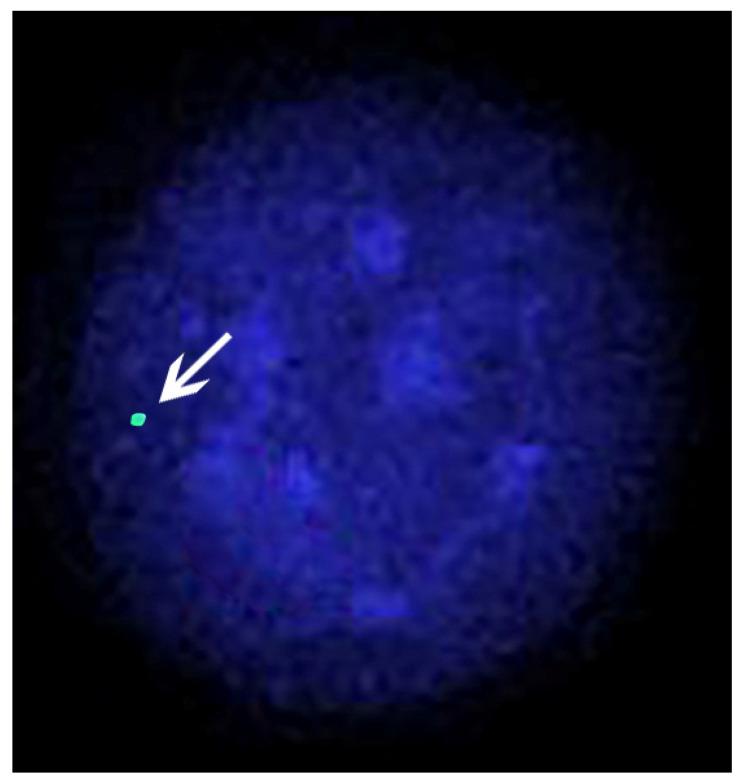
The interphase nucleus of a river buffalo female affected by X-chromosome monosomy, and showing only one FITC signal (arrow) after FISH mapping with a specific marker (PGK) of the X chromosome.

Four cases of X-chromosome monosomy were found in river buffaloes: two were in the Murrah breed [100,101], and two were in the Italian Mediterranean breed [97,98]. In all cases, the individuals were affected by gonadal dysgenesis (Table 3). In Figure 6, an interphase nucleus of a river buffalo female affected by X-chromosome monosomy is shown.

As shown in Table 3, most sex chromosome abnormalities are XX-XY mosaicisms (freemartin). This type of chromosomal abnormality occurs when placental anastomoses originate between heterosexual twins. Approximately 90% of these twins are freemartin in cattle, and most females are generally sterile as a result of severe damage to their internal sex organs [3,102]. These deleterious effects on internal sex adducts are because of the Y chromosome and the male-determining regions occurring in the blood cells. This phenomenon comprises the following two subsequent events: (1) placental anastomosis occurs 20 days before sex differentiation, and (2) male cell differentiation occurs one week earlier in males than in females [103]. For this reason, deleterious effects on the internal sex organs are much more frequent in female twins than in males, although some damage to male organs has also been reported in male twins [104,105]. However, the phenomenon of freemartinism is related to the percentage of twins being relatively high in cattle (varying from 2 to 4% in dairy breeds, reaching higher values (6%) in older cows) [106]. In buffaloes, the percentage of twins is meager (0.14%; [107]) but, unlike cattle, most females are found to be freemartin from a single birth. Indeed, as shown in Table 3, only four twins (two births) were born in both males and females, and all remaining cases were only female freemartins from a single birth. This finding means that the males died during early embryonic life and were adsorbed.

These data lead us to two important conclusions: (1) in buffaloes, the actual percentage of twins (at least in early embryonic life) is much higher than that observed; and (2) since most of the cases are from single birth, the breeder cannot realize that the female is a freemartin case, and keeps it in the farm as a normal individual for years, causing severe economic damage to the farm. Only after the female reaches reproductive age with no pregnancy, despite the presence of a bull, can veterinarian controls (e.g., rectal palpation or eco-graphic analyzes) and proper cytogenetic and genetic controls (e.g., PCR with specific male markers) be used to reveal freemartinism. However, male traits (e.g., prominent withers, larger horn base circumference, tight pelvis) were observed (Table 3); thus, both breeders and veterinarian doctors should pay careful attention to the external body traits of females at a young age in order to perform an earlier diagnosis of freemartinism via cytogenetic or molecular (PCR) analyses.

At least for the cytogenetic controls performed in the Italian Mediterranean breed of all studied females with reproductive problems, ~20% were found to be affected by sex chromosome abnormalities [97,98] (Table 3). These data underline the necessity of carefully investigating all females with reproductive problems in order to drastically reduce the economic damage caused by keeping females who will never produce calves or lactations for years. In this case, a minimum of 50 cells must be studied for each animal with reduced fertility using the C-banding technique, because sex chromosomes can be easily detected using this technique [11,57]. Indeed, the X chromosome is the largest acrocentric chromosome with the largest centromeric positive C-band, with an additional one located proximally (Figure 1). At the same time, the Y chromosome appears as completely heterochromatic (C-band positive) or with a positive C-band only situated distally (Figure 1), while all remaining acrocentric chromosomes are C-band positive in the centromeric regions [57,58] (Figure 1). Additional banding (preferably R-banding) and FISH (if necessary) techniques must be applied to animals affected by numerical autosome and structural chromosome abnormalities involving autosomes and sex chromosomes. However, using C- and R-banding techniques for all males addressed to reproduction and females with reproductive problems is highly suggested.

Chromosomes have also been used to establish the level of genome stability (or instability) in the cells of animals exposed to mutagens in vivo or in vitro, by using cytogenetic tests such as the CA test (chromatid or chromosome breaks), SCE test (sister chromatid exchange), MN test (micronuclei), comet assay test, and telomere test by RLTL. Some of these techniques have been applied in river buffalo, and are reported in Table 4; the type of test and the main results achieved are also indicated.

**Table 3:** Cases of river buffaloes (breed/country) per year affected by chromosomal abnormalities, their phenotypic effects, and references.

Breed/Country	Chromosome Abnormality	Sex	Phenotypic Effects	References
Murrah/India	XX/XY mosaicism (Freemartin)	F	Cell mosaicism found in a a triplet birth; female with internal sex damage	[108]
Murrah/India	X-trisomy (2n = 51, XXX)	F	Normal body conformation, reduced fertility (only two lactations in 10 years)	[99]
Murrah/India	X-trisomy (2n = 51, XXX)	F	Sterile (damages to internal sex structures)	[96]
Murrah/India	X-monosomy (2n = 49, X)		Gonadal disgenesis (sterility)	[100]
Murrah/India	X-monosomy (2n = 49, X)		Gonadal disgenesis (sterility)	[101]
Italian Mediterranean/ Italy	X-monosomy (2n = 49, X)	F	Normal body conformation and external genitalia; small uterine body; ovaries not detectable; sterile	[109]
Ital. Mediterranean/ Italy	XY-sex reversal (2n = 50, XY)	F	Slight hypoplasia of derivative Muller’s ducts, as well as small cervix uteri and ovary structure; sterile	[110]
Ital. Mediterranean/ Italy	X-trisomy (2n = 51, XXX)	F	Normal weight but presence of male traits (prominent withers, large horn base circumference); normal vagina and clitoris; atrophy of internal sex adducts; sterile	[97]
Ital. Mediterranean/ Italy	XY-sex reversal (2n = 50, XY)	F	Normal weight with presence of male traits (prominent withers, large horn base circumference); close vagina; absence of internal sex adducts	[97]
Ital. Mediterranean/ Italy	XX/XY mosaicism (Freematrtin) twin	F	Normal body conformation and external genitalia; atrophy of internal sex adducts; sterile	[111]
Ital. Mediterranean/ Italy	XX/XY mosaicism (Freemartin), twin	M	Normal body conformation and external genitalia	[111]
Ital. Mediterranean/ Italy	XX/XY mosaicism (Freemartin)	F	Presence of some male traits (tight pelvis, large horn base circumference); normal external genitalia; serious atrophy of Muller’s ducts; small ovaries; sterile	[111]
Ital. Mediterranean/ Italy	XX/XY mosaicism (Freemartin)	F	Normal body conformation; normal vulva, vagina, and clitoris; atrophy of internal sex adducts; ovaries not detectable; sterile.	[111]
Ital. Mediterranean/ Italy	XX/XY mosaicism (Freemartin)	F	Normal body conformation and external genitalia; atrophy of internal sex adducts; ovaries not detectable; sterile	[111]
Ital. Mediterranean/ Italy	XX/XY mosaicism (Freemartin) twin	M	Normal body conformation and penis; one testis much smaller than the other.	[111]
Ital. Mediterranean /Italy	XX/XY mosaicism (Freemartin) twin	F	Body conformation with some male traits (tight pelvis) and unusual horns (thin); normal vulva, vagina, and clitoris; lack of internal sex adducts; sterile.	[111]
Ital. Mediterranean/ Italy	XX/XY mosaicism (Freemartin)	F	Normal body conformation; normal vulva, vagina, and clitoris; atrophy of internal sex adducts; ovaries not detectable; sterile.	[111]
Ital. Mediterranean/ Italy	XX/XY mosaicism (Freemartin)	F	Normal body conformation and external genitalia; atrophy of internal sex adducts; sterile	[111]
Murrah/India	XXY-syndrome 2n = 50,Y, rob(X;X)	M	Testicular hypoplasia; azoospermic	[112,113]
Ital. Mediterranean/ Italy	XX/XY mosaicism (freemartin)	M	Normal body conformation and external genitalia, with one testis smaller than the other; fertile (with progeny)	[98]
Ital. Mediterranean/ Italy	XX/XY mosaicism (freemartin)	F	Normal body conformation and external genitalia; closed vagina with lack of internal sex adducts; sterile	[98]
Ital. Mediterranean/ Italy	X-monosomy (2n = 49, X)	F	Normal body conformation; clitoris larger than normal; small uterine cervix; uteri horns very thin; gonads absent; sterile	[98]
Ital. Mediterranean/ Italy	X-trisomy (2n = 51, XXX)		Normal body conformation; atrophy of internal sex adducts; sterile	[98]
Ital Mediterranean/ Italy	XX/XY mosaicism (freemartin)	F	Normal body conformation and external genitalia; atrophy of internal sex adducts; small ovaries; sterile	[98]
Ital. Mediterranean/ Italy	XX/XY mosaicism (freemartin)	F	Presence of male traits (tight pelvis) and thin horns; normal external genitalia and vagina; lack of internal sex adducts; sterile	[98]
Ital. Mediterranean/ Italy	XX/XY mosaicism (freemartin)	F	Normal body conformation and external genitalia; absence of internal sex adducts with closed vagina; sterile	[98]
Ital. Mediterranean/ Italy	XX/XY mosaicism (freemartin)	F	Normal body conformation and external genitalia; atrophy of internal sex adducts; small ovaries; sterile	[98]
Ital. Mediterranean/ Italy	XX/XY mosaicism (freemartin)	F	Normal body conformation and external genitalia; small uterine body; sterile	[98]
Ital. Mediterranean/ Italy	XX/XY mosaicism (freemartin)	F	Normal body conformation and external genitalia; small uterine body and ovaries; sterile	[98]
Ital. Mediterranean/ Italy	XX/XY mosaicism (freemartin)	F	Normal body conformation and external genitalia; small uterine body; sterile	[98]
Ital. Mediterranean/ Italy	XX/XY mosaicism (freemartin)	F	Normal vulva with large clitoris; atrophy of internal sex adducts; sterile	[98]
Ital. Mediterranean/ Italy	XX/XY mosaicism (freemartin)	F	Some male traits (pelvis slightly thin); normal external genitalia; closed vagina with absence of internal sex adducts; sterile	[98]
Ital. Mediterranean/ Italy	XX/XY mosaicism (freemartin)	F	Head with slight male traits, but with horns smaller than normal (horn base circumference 26 cm); uterine body incomplete; presence of a small left uterine horn without ovary; sterile	[98]
Ital. Mediterranean/ Italy	XX/XY mosaicism (freemartin)	F	Head with male traits, normal external genitalia; internal sex adducts showing a very small uterine body developed with a draft right uterine horn and atrophic uterine left horn with a draft ovary; sterile	[98]
Ital. Mediterranean/ Italy	XX/XY mosaicism (freemartin)	F	Normal body conformation and external genitalia, with a small clitoris, and atrophic internal adducts, with both uterine body and uterine horns being as fibroid cords without lumen	[98]
Ital. Mediterranean/ Italy	rob(1p;23), 2n = 50	F	Reduced fertility (only two lactations in five years); second female calf carrying the same abnormality	[98]
Ital. Mediterranean/ Italy	rob(1p;18), 2n = 50	M	Famous bull eliminated from reproduction; the abnormality was also found in some of the progeny; sperm FISH in the motile fraction sperm revealed limited effect on aneuploidy in the gametes	[94] [83]
Murrah/India	Mosaicism 20 = 50, XY/2n = 50, XY, 3q-	M	Phenotypically normal and fertile bull aged between 40 and 42 months. Not yet used for reproduction	[95]

**Table 4:** Cytogenetic test applied to river buffalo cells or sperm after in vivo (natural exposure) or in vitro exposure to potential mutagens; results and references.

Breed/Country	Cytogenetic Test	In Vivo/In Vitro Exposure	Results	Reference
Ital. Mediterranean/ Italy	SCE	In vitro	Normal SCE baseline in blood cells	[114]
Mediterranean/Egypt	Chromosome breaks, SCE	In vivo	Increased chromosomal fragility in buffaloes raised in polluted areas	[115]
Ital. Mediterranean/ Italy	SCE	In vitro	Chromosome instability induced in the X chromosome by exposure to Mitomicin C	[116]
Murah, Jaffarabadi, Italian Mediterranea/Brazil	Chromosome breaks	In vivo	Fragility in the X chromosome	[117]
Mediterranean/Egypt	Chromosome breaks, SCE, micronuclei	In vitro	Clastogenic effects of the fasciolicide drug Fasinex	[45]
Ital. Mediterranean/ Italy	DNA polymerase alpha inhibition by aphidicolin	In vitro	Fragile sites mainly found in specific chromosomes	[118]
Ital. Mediterranean/ Italy	Chromosome breaks, SCE, aneuploidy	In vitro	Chromosomal instability in calves with limb malformation (transversal hemimelia—TH)	[46]
Murrah/India	DNA sperm fragmentation	In vivo	Normal baseline of buffalo DNA sperm fragmentation	[119]
Ital. Mediterranean/ Italy	Chromosome breaks, SCE	In vivo exposure to dioxins	Chromosome fragility	[42]
Murrah/India	DNA sperm fragmentation	In vitro (titanium oxide (TiO2) nanoparticles (NPs exposure)	Cytotoxic effect on buffalo spermatozoa	[44]
Ital. Mediterranean/ Italy	SCE	In vitro	No chromosomal fragility in cells exposed to furocumarin extracts	[43]
Italian Mediterranean/ Italy	Chromosome breaks, SCE, micronuclei, RLTL	In vivo	No chromosomal fragility in blood cells of buffaloes raised in urban and rural areas	[41]

## 4. Molecular Cytogenetics

Molecular cytogenetics was first applied to human chromosomes when specific DNA probes were hybridized using a new and advanced technique—fluorescence in situ hybridization (FISH) [120,121,122]. The DNA probes are made with cDNA, which is only applicable when the target gene is multicopy. Nevertheless, more generally, they may also be made with genomic DNA of different sizes, such as cosmids (DNA insert sizes of 20–40 kb) or, more commonly, bacterial artificial chromosomes (BACs; DNA insert sizes of 100–300 kb). Therefore, with these DNA probes, stronger hybridization signals are able to be visualized. Depending on the type of probe (mapping a specific chromosome region with the classical FISH), entire chromosomes, chromosome arms (painting probes; Zoo-FISH), specific chromosome regions, or whole chromosomes will be fluorescent in the dark field (Figure 3c–e). Chromosome painting probes are generally obtained by cell sorter chromosomes or by chromosome microdissection. The only (partial) chromosome libraries available in river buffalo are those obtained via chromosome microdissection [25,123].

Generally, the FISH technique allows us to (1) physically map genetic loci on specific chromosome regions [10,124,125]; (2) compare entire chromosome regions between related and unrelated species (Zoo-FISH) [20,21,22,23,24,25,26,27,28,126]; (3) establish the gene order along the chromosomes using detailed FISH-mapping data, which are adequate to detect chromosomal abnormalities (e.g., inversions, deletion, reciprocal, and Robertsonian translocations) (reviewed in [3,4,5,6,7]), or to compare different species on the basis of the gene order in homologous chromosomes or chromosome regions [24,32,34,35,36,127,128]; and (4) detect chromosome aneuploidy or trisomy in both metaphase [97,109] and interphase nuclei of somatic (Figure 6) and germinal cells [4,83,129].

The FISH technique was first applied to domestic animals to physically map the beta-casein gene on cattle chromosomes [124], or to correctly identify a chromosomal abnormality in cattle [130]. Comparative FISH mapping was first applied to domestic bovids to map the major histocompatibility complex (MHC) locus on cattle and river buffalo [67], and the omega and trophoblast interferon genes in cattle, river buffalo, sheep, and goats [66]. In these studies and others that followed, the high degree of chromosome and loci conservation among related bovids was confirmed [32,33,57,68]. The FISH technique was used to physically map several loci in river buffalo chromosomes, especially after the publication of the standard river buffalo karyotype [47]. The most complete cytogenetic map for the river buffalo was reported in [10], where 308 loci were mapped. However, the FISH technique has also been used in river buffalo to (1) compare human and river buffalo genomes via Zoo-FISH, using human chromosome libraries as probes, which is helpful in detecting the human chromosome regions conserved in river buffalo chromosomes [23]; (2) resolve evolutionary events in which the river buffalo was differentiated from other bovids [35,57,68,88]; (3) correctly identify the river buffalo chromosomes involved in chromosomal abnormalities [93,94,109,110,131]; (4) support the radiation hybrid (RH) maps performed in river buffalo [132,133,134]; and (5) check the fertility degree of bull semen carrying a chromosomal abnormality via sperm FISH [83].

As reported previously, Zoo-FISH is helpful for checking the conserved chromosome regions between related and unrelated species [20,21,22,23,24,25,26,27,28]. Still, the gene order between species is unknown, especially between distantly related mammals (i.e., humans–bovids). This issue can be easily resolved via FISH mapping of individual loci along the chromosomes, allowing us to reveal the correct gene order in chromosome regions between related and unrelated species in order to precisely establish conserved chromosome regions in several species, including the water buffalo [35,57,68,88].

## 5. Conclusions

The water buffalo remains of interest for its strategic and economic importance to the large human population raising this species. The chromosomes remain a fascinating material to differentiate not only the various species of buffalo in the world, but also the results of crosses between different subspecies. From an evolutionary point of view, cytogenetic studies of wild buffalo (*B. arnee*) should be undertaken in order to better establish the origins of various species of water buffaloes. Crosses between river and swamp buffaloes are important to noticeably increase milk production, although problems can arise as a result of the limited food available in swamp buffalo farms supporting higher milk production, as well as reproductive problems during the meiosis of hybrids with 49 chromosomes, as discussed above. Cytogenetic controls—at least for all males addressed to reproduction and females with reproductive problems (e.g., lack of estrus in reproductive age, larger than normal interbirth interval)—should be implemented to add genetic and economic value to the water buffalo food chain. Collaboration between breeders, veterinarian doctors, and cytogenetic labs from various countries is essential in order to reach this goal. More progress in the genetic improvement of buffalo cytogenetics can be obtained using the CGH array, as done in cattle, to reveal genomic DNA losses during the formation of chromosomal abnormalities. It is still essential to extend the water buffalo cytogenetic maps in order to noticeably increase the number of mapped loci (type I and type II). Such an extension will better anchor hybrid radiation maps to specific chromosome regions, and help to allocate genetic sequences of milk and meat production genes.

## Figures and Tables

**Table 1:** Diploid number (2n), fundamental number (FN), and cattle homologous chromosomes (ancestral bovid karyotype) involved in the autosomal biarmed pairs of both African (*Syncerus*) and Asiatic (*Bubalus*) buffaloes according to [47,52,62,65].

Buffalo	Genus	Species	Sub-Species	2n/FN	Biarmed Pairs (Cattle Homologous Chromosomes Are Reported between Parentheses)					
African	*Syncerus*	*caffer*	*caffer*	52/60	1(1;13)	2(2;3)	3(5;20)	4(11;29)		
	*Syncerus*	*caffer*	*nanus*	54/60	1(1;13)	2(2;3)	3(5;20)			
Asiatic	*Bubalus*	*bubalis*	*bubalis*	50/60	1(1;27)	2(2;23)	3(8;19)	4(5;28)	5(16;29)	
	*Bubalus*	*bubalis*	*carabenensis*	48/58	1(5;28;7) *	2(1;27)	3(2;23)	4(8;19)	5(16;29)	
	*Bubalus*	*mindorensis*		46/58	1(5;28;7) *	2(1;27)	3(2;23)	4(8;19)	5(4;14)	6(16;29)
	*Bubalus*	*depressicornis*	*Depressicornis*	48/60	1(1;27)	2(2;23)	3(8;19)	4(5;28)	5(11;20)	6(17;15)
	*Bubalus*	*depressicornis*	*quarlesi*	48/60	1(1;27)	2(2;23)	3(8;19)	4(5;28)	5(11;20)	6(17;15)

* Centric fusion + tandem fusion.

## Data Availability

Data are contained within the article.
